# An internal hernia projecting through a mesenteric defect following laparoscopic-assisted partial resection of the transverse colon to the lesser omental cleft: report of a case

**DOI:** 10.1007/s00595-012-0264-z

**Published:** 2012-07-21

**Authors:** Shinsuke Masubuchi, Junji Okuda, Keitarou Tanaka, Keisaku Kondo, Keiko Asai, Hajime Kayano, Masashi Yamamoto, Kazuhisa Uchiyama

**Affiliations:** Departments of General and Gastroenterological Surgery, Osaka Medical College Hospital, 2-7 Daigaku-machi, Takatsuki, Osaka 569-8686 Japan

**Keywords:** Internal hernia, Laparoscopic colectomy, Mesenteric defect, Lesser omental cleft

## Abstract

We herein report a case of an internal hernia projecting through a mesenteric defect following laparoscopic-assisted colectomy to the lesser omental cleft in a 61-year-old female. We performed laparoscopic-assisted partial resection of the transverse colon to treat transverse colon cancer. Three years and 6 months after the operation, the patient developed a bowel obstruction requiring surgical intervention. When we observed the intraperitoneal space under laparoscopy, we determined that the small intestine had passed into the bursa omentalis through the mesenteric defect. Additionally, an abnormal opening of the lesser omentum was present with a portion of the small intestine escaping into the space inferior to the liver. We performed reintegration of the escaped bowel and closed the mesenteric defect laparoscopically. This is the first case of an internal hernia projecting through a mesenteric defect following laparoscopic-assisted colectomy that we have experienced out of more than 2400 cases. Further research is needed to identify the patients who would benefit from the closure of mesenteric defects during laparoscopic-assisted colectomy.

## Introduction

In recent years, as laparoscopic surgery has developed, this surgical technique has become a standard operative method for the treatment of colorectal cancer. However, there is no consensus concerning whether to close mesenteric defects after laparoscopic-assisted colectomy (LAC). In general, we close mesenteric defects during open colectomy. However, numerous reports indicate that mesenteric defects are often not closed during laparoscopic surgery and we generally follow this practice. We herein report a case of an internal hernia projecting through a mesenteric defect following laparoscopic-assisted partial resection of the transverse colon to the lesser omental cleft.

## Case presentation

**Fig. 1:**
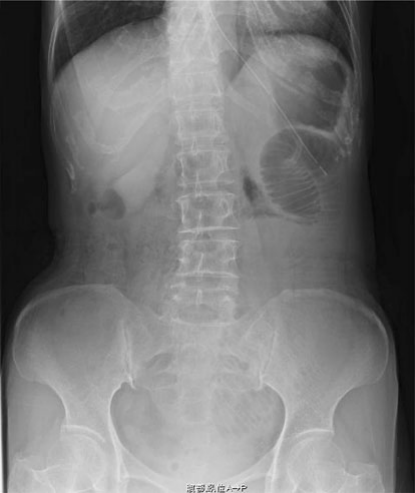
Plain abdominal radiograph. The air-fluid levels in the* left upper quadrant*

**Fig. 2:**
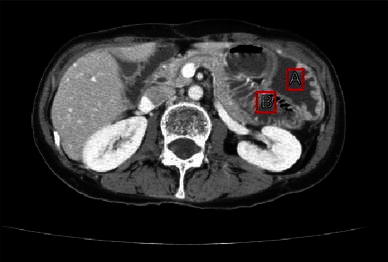
Abdominal computed tomography. Caliber changes are seen in the small bowel. *A* stomach; *B* ileum

A 57-year-old female was admitted to our hospital for the surgical treatment of transverse colon cancer. A colonoscopic examination demonstrated the presence of a 2.3 × 2.0 cm protruded lesion in the left transverse colon. A biopsy was performed and well-differentiated adenocarcinoma was diagnosed. Laparoscopic-assisted partial resection of the transverse colon was performed. We ligated the sub-middle colic artery feeding the lesion and kept the middle and left colic arteries (D3 lymph node dissection). After mobilization of the splenic flexure was performed, the bowel loop was delivered under a wound protector through a 5-cm midline incision above the navel. Division of the marginal vessels and functional end-to-end anastomosis was performed extracorporeally with linear staplers (PROXIMATE Linear Cutter 75; Ethicon Endo-Surgery, Cincinnati, OH). The mesenteric defect resulting from the bowel resection was not closed. After closure of the minilaparotomy was completed, the anastomosis and mesesnteric defect were checked laparoscopically and found to be normal without any internal hernias. The tumor was diagnosed pathologically as a T2, N0, M0, stage I (TNM classification) adenocarcinoma. The patient showed no signs of recurrence following the operation. Three years and 6 months after the operation, the patient developed a bowel obstruction. She had nausea, vomiting and abdominal distension. A plain abdominal X-ray revealed the air-fluid levels in the left upper quadrant (Fig. 1). Computed tomography revealed caliber changes in the ileum (Fig. 2). This finding was initially thought to be compatible with a diagnosis of adhesive small bowel obstruction. Tapering of the ileum was seen under the navel. Because decompression with a long intestinal tube failed to resolve the bowel obstruction, surgery was thus performed.

**Fig. 3:**
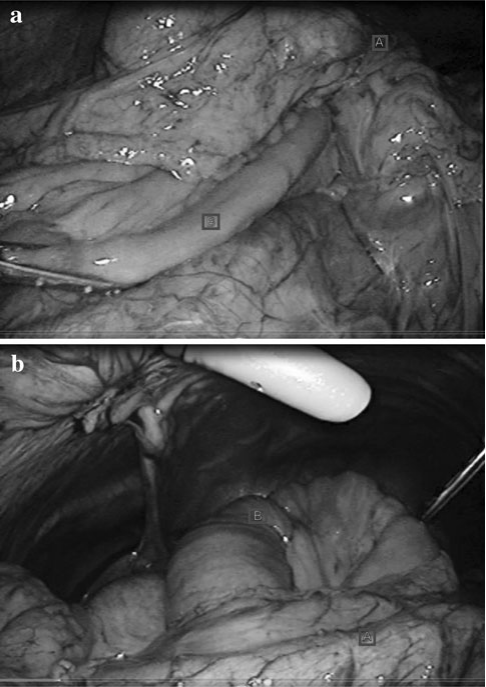
**a**, **b** Operative findings. **a** The ileum is herniated through the mesenteric defect. *A*, anastomosis, *B*, herniated ileum. **b** The small intestine passed into the bursa omentalis through the mesenteric defect and an abnormal opening of the lesser omentum was present with a portion of the small intestine escaping into the space inferior to the liver. *A* Stomach, *B* herniated ileum

**Fig. 4:**
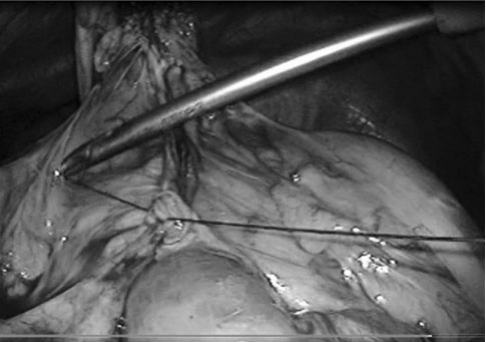
Closure of the mesenteric defect

We initially chose to treat the obstruction laparoscopically. When we observed the intraperitoneal space, we found that the small intestine had passed into the bursa omentalis through the mesenteric defect. Additionally, an abnormal opening of the lesser omentum was present with a portion of the small intestine escaping into the space inferior to the liver (Fig. 3a, b). We performed reintegration of the escaped small intestine and closed the mesenteric defect (Fig. 4). We were able to complete the operation laparoscopically. The patient’s postoperative course was uneventful. At the 9-month follow-up examination, no clinical or radiographic evidence of the cancer or the internal hernia was observed.

## Discussion

The use of LAC has become widespread in recent years, and the results obtained with this technique have been reported to be equivalent to those of open colectomy [1] Therefore, LAC has become a standard operative approach. Previous studies have reported that the incidence of small bowel obstruction following laparoscopic colectomy ranges from 0.8 to 2.5 %, which is lower than that seen with open colectomy [2–5]. Mesenteric defects caused by colectomy performed to treat colorectal cancer are generally closed as part of open colectomy because the defects may cause internal hernias. However, there is no consensus concerning the need for closure of mesenteric defects following LAC [6]. Closure of mesenteric defects during laparoscopic surgery is time-consuming, technically challenging and may jeopardize the blood supply to the anastomosis. In addition, small bowel obstructions related to mesenteric defects from laparoscopic surgery are rarely reported. Alternatively, mesenteric defects may be closed in an open fashion using wound protectors. However, this procedure is unlikely to allow for safe and complete closure of defects through the small laparotomy wounds used in LAC in the majority of patients. The potential morbidity associated with closing defects, including possible injury to the bowel and vasculature, may outweigh the risk of leaving the defects open [6]. However, narrow residual defects (2–5 cm) resulting from incomplete closure may increase the risk of developing symptomatic internal hernias [7].

We have performed more than 2400 operations using LAC. We did not close mesenteric defects following LAC. The case we report here is the first case of an internal hernia resulting from a mesenteric defect following LAC at our institution. To the best of our knowledge, there are only 12 cases of internal hernias occurring after LAC reported in the literature [2, 4–11]. We were unable to identify any reports of internal hernias anastomose occurring after protectomy. We hypothesize that anatomical differences may explain this difference. When we draw the colon outside of the body to separate and anastomose it, there is a possibility that an internal hernia may occur because a sufficient length of the colon creates space for the small bowel to fall into the mesenteric defect. However, in cases of proctectomy, there is no space for the small bowel to fall into the mesenteric defect posteriorly.

It has been reported that the incidence of internal hernias such as Petersen’s hernias increases after laparoscopic gastrectomy with Roux-en-Y reconstruction for gastric cancer and laparoscopic gastric bypass for morbid obesity because patients who undergo these operations tend to exhibit body weight loss [12–15]. It has been suggested that body weight loss leads to increases in the size of existing small mesenteric defects due to thinning of the mesentery [12–15]. Moreover, laparoscopic surgery reduces the adhesion of the small intestine and may result in greater mobility of the small bowel, further increasing the risk of herniation compared with open surgery [12, 14, 16]. Excessive body weight loss due to colectomy is rare; however, we suppose that the adhesion of the small intestine is reduced by laparoscopic surgery. A slender mesentery in a thin patient can pass into a mesenteric defect easily, further increasing the risk of herniation. It is necessary to be concerned about internal hernias resulting from mesenteric defects in thin patients. In the case of a very thin patient, closing a mesenteric defect laparoscopically during the first operation may prevent the development of an internal hernia. We fill mesenteric defects with the greater omentum when possible; however this was not performed in this case. Although this patient did not exhibit excess body weight loss, she was thin originally (body mass index: 16 kg/m^2^). Additionally, we suppose that filling mesenteric defects with the greater omentum instead of closing them may possibly prevent the development of internal hernias. In our facilities, suturing to reinforce anastomotic regions during laparoscopic anterior resection is performed routinely. By experiencing many cases, we were able to master the suturing technique used in laparoscopic surgery. We think that mesenteric defects can be closed safely, exactly and quickly in thin patients. Although there are almost no opportunities to suture laparoscopically, as compared with gastric surgery and laparoscopic colorectal surgery, it is important to master the suturing technique under laparoscopy. Moreover, lesser omental hernias are very rare causes of internal hernias. No reports have identified the causes of lesser omental clefts, although there are various hypotheses involving mechanisms, such as congenital susceptibility, inflammation or trauma [17–21].

Because small bowel obstructions resulting from internal hernias projecting through mesenteric defects following LAC are rare, there is no need to close mesenteric defects following LAC in all cases. However, because reoperation is required for treatment, further research is needed to identify the patients who would benefit from closure of mesenteric defects during LAC. We will incorporate the following procedures into our basic policy: we will assess mesenteric defects during the final stages of surgery, and if the small intestine has passed into the space, we will perform reintegration of the escaped small intestine and fill the space with the greater omentum. In cases of thin patients, it may therefore be better to close mesenteric defects laparoscopically.
